# Supplementary material for: Radiographic interpretation using high-resolution Cbct to diagnose degenerative temporomandibular joint disease
Source: PLoS One. 2021 Aug 10;16(8):e0255937. doi: 10.1371/journal.pone.0255937 (PMC8354480; doi:10.1371/journal.pone.0255937)
Supplement: Attachment — Submitted filename: Reply to the reviewers v3.0.docx [file pone.0255937.s003.docx]

We thank you to all the three reviewers and editors that spent time and effort helping to improve this manuscript. We certainly have increased the quality of our data after your suggestions and comments.

Sincerely,

Authors.

**Reviewer #2:**

We thank you so much for your comments and observations. They improved the quality of our work. We greatly appreciate your time and careful analysis of our study, and we hope to provide the answers to your questions/suggestions.

**1. Comment 5. The word "expert" is a difficult one to quantify and so should be avoided. When referring to the individuals who viewed the CBCT images, I would suggest you the term** **"board-certified oral and maxillofacial radiologist".**

We have addressed the changes as requested. We are sorry that we have not corrected this properly in the previous review.  **2. Comment 9. I would remove any and all references to dosimetry since i) it was never done; and ii) you cannot apply the dosimetry of one system to another, particularly if the center of the imaging volume is not the same. I believe I made mention of this in my original comments.**

We agree with the reviewer, we have added as a limitation previously, but we agree that using this in our methodology can lead the readers to the wrong perception. Therefore, as we have not performed a dosimetry study, we have removed the information from our manuscript, as suggested.  **1. The use of English in this revision reads more poorly than the initial submission. It requires some work.**

We did a general English correction in the entire paper with our department's native English speaker staff; the changes are tracked.

**2. Abstract. 'k' is Cohen's kappa? If 'yes', then "kappa" should be spelled out (with the name Cohen) or the appropriate Greek letter should be used.**

Thank you for this critical observation. We have corrected this not only in the abstract but in the entire manuscript as well.  **3. If you tell us the voxel is 0.08 mm, you should add a word or phrase to indicate that the voxel is isotopic since you only give 1 dimension of the 3.**

Thank you. In this revised version, we added this information.  **4. There should be alignment between the Objective/Aim and what is being concluded. This does not occur in the Abstract.**

We wrote the conclusion again, and we hope to be now better aligned with our aim.  **5. Introduction. CBCT imaging is not a "dental research" tool, so the first sentence in the second paragraph is a strange assertion.**

We changed this paragraph as follows: "In dentistry, CBCT is the exam of choice to evaluate many bone-related diseases."

**6. In the last paragraph of the Introduction, an Aim or Objective, or better yet, a hypothesis should be included. Providing insight" is not scientific.**

We agree; we have now stated our hypothesis.

**7. Materials and Methods. What is "STROBE"?**

We have added this information in the material and methods section. (Strengthening the Reporting of Observational Studies in Epidemiology (STROBE))
 **8. Again, reference is incorrectly made to 43 uSv as the dose for each TMJ in the Materials and Methods section in the paragraph "High-resolution CBCT radiation information". For the reasons I gave in the original review about dosimetric calculations, 43 uSv is misleading and erroneous. I don't know why such references continue in this revised version.**

Thank you again for this observation; we now have removed this information since this was not our aim, and we did not perform a dosimetry study. However, we also have included the manuscript provided by you from the Toronto group in our discussion as additional information.

**9. To be clear, this is the Department of [MEDICAL] Radiology at the University of Michigan?**

This was the Department of Periodontics & Oral Medicine at the University of Michigan – School of Dentistry. We have corrected this in our manuscript. Thank you for this observation; we have not noticed this typo before because both professors (radiologists) teach radiology at the school. **10. The references numbers do not align with the correct references in the text.**

We did an overall review of the references/numbers. The problems occurred during the track version changes / clean version and reference manager.

**11. I am still suspicious of the validity of the radiologic observations. In Figure 4A, the "Cyst" is not round or hydraulic or smooth (as cysts should be). This is not a cyst but likely a marrow space like the other similarly appearing marrow spaces in the condylar neck.**

Thank you for your observation; you are right. I have consulted the two radiologists who agreed with your comments. For this reason, I have chosen another case to illustrate the radiologist's findings.

**12. In Figure 4B, how can an erosion be located along the anterior surface of the condylar head since there is no biomechanical loading in this area.**

I'm sorry, I have added the arrow in the wrong direction. As I do not have the original PowerPoint to modify the arrow position, I choose another image to replace 4B. Thank you. **13. Figure 4E is a nice example of a subchondral cyst; it is not an erosion because I can resolve a cortex that is intact.**

After consulting the radiologists, they also agree with you. For this reason, I have chosen another case to illustrate the erosion in the control group.
